# The impact of allergic rhinitis on nasopharyngeal microbiota and metabolomics in children with severe adenoid hypertrophy

**DOI:** 10.3389/fmed.2026.1895589

**Published:** 2026-10-15

**Authors:** Binru Wang, Tian Chen, Shan Gao, Hui Jin, Gengtian Liang, Guoqing Gong, Longkai Xiao

**Affiliations:** 1 Department of Otolaryngology Head and Neck Surgery, Wuhan Third Hospital (Tongren Hospital of Wuhan University), Wuhan, China; 2 Department of Otolaryngology-Head and Neck Surgery, Renmin Hospital of Wuhan University, Wuhan, Hubei, China; 3 Department of Anesthesiology, Wuhan Third Hospital (Tongren Hospital of Wuhan University), Wuhan, China

**Keywords:** adenoid hypertrophy, allergic rhinitis, children, metabolites, microbiota

## Abstract

**Introduction:**

The impact of allergic rhinitis (AR) on adenoid hypertrophy (AH) in pediatric patients is unclear. This study aims to investigate the effects of AR on the nasopharyngeal microbiota and metabolomics of children with AH.

**Methods:**

16S rDNA sequencing and untargeted metabolomics were used to evaluate the nasopharyngeal microbiota and metabolomics characteristics of 9 children with AH complicated with AR (AH-AR group) and 9 children with AH without complicated AR (AH-nAR group).

**Results:**

The 16S rDNA sequencing results showed that compared with the AH-nAR group, the diversity of nasopharyngeal microbiota in the AH-AR group significantly increased, mainly manifested by a significant increase in Fusobacterium, Actinomyces, and Veillonella genera. Further metabolomics showed that compared with the AHnAR group, 140 metabolites were significantly reduced and 115 metabolites were significantly increased in the adenoid surface secretion samples of the AH-AR group. KEGG enrichment analysis showed significant changes in 12 metabolic pathways in the AH-AR group. In addition, Spearman correlation analysis revealed a significant correlation between altered bacterial genera and metabolites.

**Discussion:**

AR may promote AH in children through the nasopharyngeal microbiota and metabolites.

## Introduction

Adenoid hypertrophy (AH) is a pathological hyperplasia of the adenoids, which can cause symptoms or diseases such as mouth breathing, sinusitis, otitis media, asthma, speech problems, sleep snoring, etc. Some AH patients may even cause sleep apnea, “adenoid appearance”, obesity, cardiovascular disease, cognitive dysfunction, etc (1). The adenoid body (nasopharyngeal tonsil) is a lymphoid tissue block located at the junction of the top and back walls of the nasopharynx. It is the earliest immune organ to come into contact with respiratory and digestive antigens and plays an important role in the development of children's immune system (2). AH is a natural response to increased immune activity in early life, which is associated with abnormal immune responses, infections, environmental exposure, and hormonal or genetic factors. The specific mechanism is not yet fully understood. Allergic rhinitis (AR) is a nasal mucosal hypersensitivity reaction mediated by immunoglobulin E (IgE) after exposure to allergens, characterized by runny nose, nasal congestion, nasal itching, and continuous sneezing. AR is a globally significant public health, medical, and economic issue that affects approximately 2%–25% of children (3, 4). The same child often has both AH and AR diseases at the same time, and the proportion of AH children with AR (about 59.3%) is significantly higher than that of children without AR (34.7%), indicating that specific IgE-mediated immune reactions may make children susceptible to AH (5, 6). AR may be a risk factor for AH, and the impact of allergic reactions on the clinical manifestations of AH children should be taken seriously.

The prevalence of AH ranges from 12% to 34.46% (7, 8). The most common treatment for grade III and IV AH is adenoidectomy, but not all children with severe AH require surgical treatment. Although surgical treatment is more direct and effective than drug therapy, it carries risks such as excessive bleeding and infection. Moreover, after adenoidectomy, there will be regrowth, with approximately 13.3%–14.5% of children undergoing secondary adenoidectomy (9, 10). Colavita et al. suggested that adenoidectomy does not have significant benefits for 80% of children with AR and AH (11). Drug anti-allergic treatment can eliminate local inflammation, improve nasal symptoms, and prevent adenoid hyperplasia again. Considering the high clinical demand for adenoidectomy, but with many complications and the disadvantage of easy recurrence of hyperplasia, exploring the impact of AR on AH in children has significant clinical guidance significance.

The popular 16S rDNA sequencing analysis technology in recent years has overcome the limitations of conventional bacterial culture and has become a new method for microbial research. Pharyngeal or nasopharyngeal swab samples are a convenient, fast, non-invasive detection method that remains relatively stable over time, making them particularly suitable for microbial community testing in infants and children. Through 16S rDNA sequencing analysis of the microbiota in nasopharyngeal swabs, it was found that there is a certain correlation between the gut microbiota and pediatric OSA (12). Throat swab analysis of oral microbiota showed that there are certain differences in the oral and nasal microbiota of children with OSA, which may cause varying degrees of tonsillar hypertrophy or AH in children (13). Teng et al. found through microbiome research that the relative abundance of the Haemophilus genus (members of the Proteobacteria phylum) increased in pediatric patients with AH and tonsillar hypertrophy, while the relative abundance of the Actinobacteria genus (members of the Actinobacteria phylum) decreased (14). However, there was no significant difference in microbial abundance between children who underwent adenoidectomy with tonsillectomy and healthy control children. This microbiome analysis provides new insights into the occurrence, development, and influencing factors of AH in children.

This study analyzed the microbiome profile of nasopharyngeal swabs from children with AH using 16S rDNA sequencing technology, and compared the differences in the microbiome profile of nasopharyngeal swabs between children with simple severe AH and those with AR, further exploring the correlation between the microbiome and metabolites in the nasopharynx of AH children, and elucidating the effect of AR on the microbiota and metabolites in the nasopharynx of AH children, providing new ideas for the etiology and treatment of AH in AR children.

## Materials and methods

### Inclusion and exclusion criteria

Collect AH patients who visited the Department of Otorhinolaryngology Head and Neck Surgery at Wuhan Third Hospital from August 2024 to November 2024, and divide them into two groups based on whether they have AR: AH without complicated AR group (AH-nAR group), with a total of 9 cases; AH complicated with AR group (AH-AR group), a total of 9 cases. Record the following clinical information of all patients: (1) age, gender, BMI (kg/m^2^); (2) measure the level of complete blood cell count+CRP (CBC+CRP); (3) measure the serum total IgE (tIgE) level; (4) perform a fiberoptic nasal endoscopy examination. (5) perform the Reflective Total Nasal Symptom Score (rTNSS).

Inclusion criteria: The child was determined to have grade IV AH through fiberoptic laryngoscopy examination, and diagnosed with AR according to the guidelines for AR and its impact on asthma (ARIA) (3, 15).

Exclusion criteria (16): (1) suffering from tonsillar hypertrophy, acute tonsillitis, or severe oral diseases (such as abscess or fistula, oral mucosal lesions, etc.); (2) suffering from acute and chronic respiratory and digestive system diseases within the past 3 months; (3) suffering from systemic diseases (including inflammation, metabolism, tumors, genetics, autoimmune diseases); (4) received nasal, oral, pharyngeal, and laryngeal procedures or treatments within the past 4 weeks; (5) have used nasal steroids, antihistamines, or leukotrienes in the past 4 weeks; (6) have used anti-inflammatory drugs or immunosuppressants that affect the microbiota and metabolism in the past 3 months; (7) smoking members in the family; (8) having specific dietary habits; (9) domestic pets.

This study was approved by the Medical Ethics Committee of Wuhan Third Hospital (No: 2025−003) and adhered to the principles of the Helsinki Declaration. All parents or guardians had signed the informed consent form.

### Whole blood cell count detection

Perform CBC + CRP testing before collecting nasopharyngeal swabs from the patient. The parameters include white blood cell count (WBC), red blood cell count (RBC), hemoglobin (Hb), hematocrit (HCT), mean corpuscular volume (MCV), mean corpuscular hemoglobin (MCH), mean corpuscular hemoglobin concentration (MCHC), red blood cell distribution width (RDW), platelet count (PLT), mean platelet volume (MPV), platelet distribution width (PDW), platelet specific volume (PCT), large platelet ratio (LPR), neutrophil percentage (N), neutrophil absolute count (ANC), lymphocyte percentage (L), lymphocyte absolute count (ALC), monocyte percentage (M), absolute Mononuclear cell count (AMC), percentage of eosinophils (E), absolute eosinophil count (AEC), percentage of eosinophils (B) And absolute count of eosinophils (ABC). Among them, the parameters related to red blood cells are RBC, Hb, HCT, MCV, MCH, MCHC, and RDW; the parameters related to white blood cells include WBC, N, ANC, L, ALC, M, AMC, E, AEC, B and ABC, while the parameters related to platelets include PLT, MPV, PCT and PDW.

### Conduct fiberoptic nasal endoscopy examination

Nasal endoscopy is the “gold standard” for evaluating AH (17). According to the “Chinese guideline for the diagnosis and treatment of childhood obstructive sleep apnea (2020)” (15), adenoids are divided into 4 degrees by percentage through fiberoptic nasal examination: degree I, 0-25%; II degree, 26%∼50%; Grade III, 51%∼75%; Grade IV, ranging from 76% to 100%, with Grade III and IV being pathological obstructions in clinical practice.

### Conduct reflective total nasal symptom score (rTNSS)

RTNSS is a scoring system that assesses the severity of nasal symptoms (including runny nose, nasal congestion, nasal itching, and sneezing) within the past 12 hours. Each symptom is scored on a scale of 0 to 3 out of 12, with higher scores indicating more severe symptoms (18).

### Blood allergen testing

Monitor serum tIgE levels. Collect fasting venous blood from two groups of patients and centrifuge at 3,000 r/min for 10 minutes (centrifuge half diameter of 8 cm), and store at −80 °C. 2103;. Use an automated detection system (Phadia, Sweden) for serum tIgE level detection. The normal reference ranges of serum tIgE for children aged 2-5 years, 6-9 years, and 10-15 years are 0.0-60.0 IU/mL, 0.0-90.0 IU/mL, and 0.0-200.0 IU/mL, respectively.

### Nasopharyngeal swab collection

The same otolaryngologist collected surface secretions of adenoids. Fasting for 8 hours before surgery and not brushing teeth within 24 hours. When the patient was under general anesthesia, use a sterile throat swab cotton swab to rotate the surface of the adenoid five times under constant pressure (to avoid damage and bleeding on the surface of the adenoid) to collect biofilm samples, and then immediately place them in a sterile tube. The swab should not contact the oral mucosa, pharyngeal mucosa, or saliva during the sampling process. Place all samples in an ice box and transfer them to a −80 °C environment for storage within 2 hours.

### DNA extraction and 16S rDNA sequencing

To maximize bacterial recovery, the swab samples were gradually raised to 4 °C and stored overnight in a 0.01 M phosphate-buffered saline (Sigma Baldric) solution. At the end of the incubation period, vortex the sample vigorously for about 5 minutes, and then centrifuge at 12.000 rfc for 5 minutes. According to the manufacturer's instructions, use the QIAamp DNA Mini Kit (Qiagen, Germany) to extract total genomic DNA from the sample. PCR products were examined on agarose gel and purified with the Omega DNA purification kit (Omega Inc., Norcross, GA, USA). Subsequently, the sequence of 16S rDNA V3-V4 region (464 bp) was amplified by PCR using 341F/805R (341F: 5'-CCTAYGGGGRBGCASCAG-3’; 805R: 5'-GGACTACHVGGGTWTCTAAT-3’). After mixing and purification, the PCR products were analyzed on the Illumina NovaSeq 6,000 platform (Beijing Biomarker Technology Co., Ltd., Beijing, China).

### Non-targeted metabolomics

Thaw the surface secretion sample of the adenoid at 4 °C, and mix 100 μL aliquots with 400 μL of cold methanol/acetonitrile (1:1, v/v) to remove proteins. Centrifuge the mixture for 20 minutes (14,000 g, 4 °C). Dry the supernatant in a vacuum centrifuge. For LC-MS analysis, transfer the supernatant to a new 96-well plate and maintain at −80 °C. Create quality control (QC) samples by mixing 10 μL of each extraction combination. Perform LC-MS analysis using a UPLC system with a reverse-phase chromatography column. Use a mobile phase consisting of solvent A (water, 0.1% formic acid) and solvent B (acetonitrile, 0.1% formic acid) for gradient elution for chromatographic separation of metabolites. Perform metabolite detection using a high-resolution tandem mass spectrometer Q-Exactive in both positive and negative ion modes. Collect mass spectrometry data using IDA mode. The preprocessing of bioinformatics analysis includes peak picking, retention time correction, isotope and adduct annotation, and metabolite annotation based on online databases such as KEGG and HMDB.

### Statistical analysis

Descriptive data were presented as SD ± mean. T-tests or Wilcoxon tests were performed to evaluate the differences in taxonomic abundance between groups. Perform chi-square test and Fisher's exact test using SPSS version 26.0. Evaluate alpha diversity by calculating the Chao1 richness index and the Shannon index. Based on the weighted unifrac distance matrix, *β* diversity analysis of nasopharyngeal microbiota was performed using PCoA. Compare bacterial abundance and diversity using one-way ANOVA. Perform a t-test on intergroup species abundance data using Metastats software. Linear discriminant analysis (LDA) effect size (LEfSe) was used to identify differences between the bacterial species between groups. For metabolomics data, t-tests or Kruskal–Wallis tests were used to analyze intergroup differences. Use Spearman correlation analysis to evaluate the correlation between bacterial genera and serum metabolites. A *p*-value < 0.05 was considered statistically significant.

## Results

### Population and clinical data

There was no significant statistical difference in gender, age, BMI(kg/m^2^) and CBC + CRP detection (*P* > 0.05), while the serum tIgE and rTNSS in the AH-AR group were significantly higher than those in the AH-nAR group (*P* < 0.05) (Supplementary Table S1).

### Analysis of surface microbiome of adenoids

In order to investigate the effect of AR on the surface microbiota of adenoids, high-throughput 16S rRNA gene sequencing was performed on surface secretion samples from 9 AH-AR groups and 9 AH-nAR groups. The results showed that there were 284 bacterial genera shared by the surface microorganisms of adenoids in the AH-nAR group and AH-AR group, each with its own unique microorganisms (Figure 1A). The alpha diversity results showed that compared with AH-nAR, the AH-AR group's species diversity index (Chao1 and Shannon) increased significantly (Figures 1B,C). Perform beta diversity analysis using principal coordinate analysis (PCoA) with the weighted UniFrac metric to detect differences in microbial community structure on the surface of various adenoids. The PCoA results showed significant differences in the surface microbial composition of adenoids between the AH-nAR group and the AH-AR group (R = 0.291, *P* = 0.003) (Figure 1D). In addition, the unweighted paired group method with arithmetic mean based on weighted UniFrac index (UPGMA) also showed significant differences in overall microbial composition between the AH-nAR group and the AH-AR group (Figure 1E).

**Figure 1:**
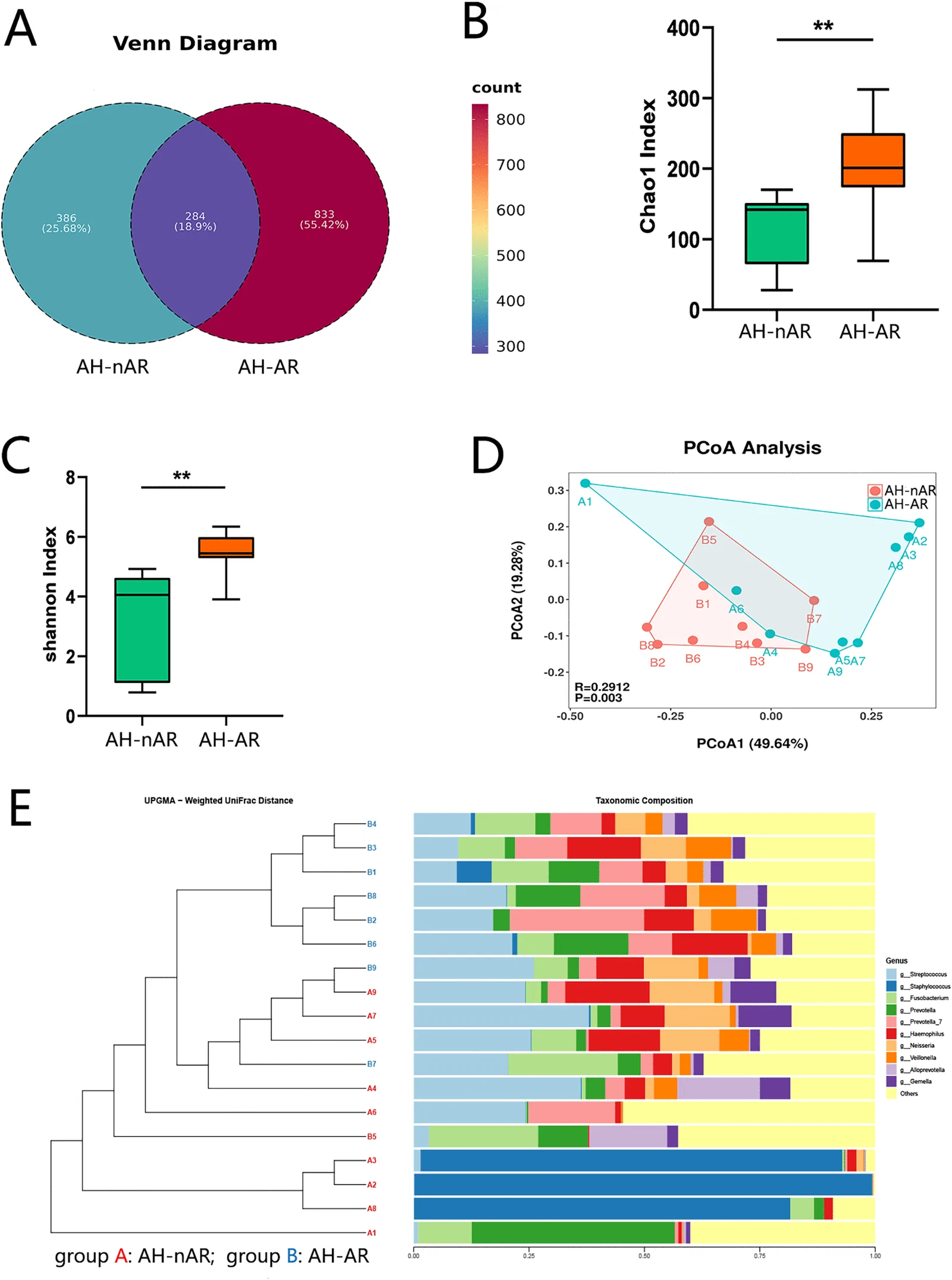
High-throughput 16S rRNA gene sequencing was employed to characterize the surface secretions of adenoids from children in the AH-nAR and AH-AR groups. **(A)** Quantity and variation of the microbiota. The quantity of microbial genera present on the adenoid surface in the two groups. **(B,C)** Alpha diversity analysis was performed to assess differences in the microbial community structure on the adenoid surface between the two groups; **(D)** Beta diversity analysis was conducted to evaluate variations in the microbial community structure on the adenoid surface between the two groups; **(E)** Unweighted Pair Group Method with Arithmetic Mean (UPGMA) clustering was used to analyze differences in the overall microbial composition on the adenoid surface between the two groups. **, *P* < 0.01.

At the phylum level, *Firmicutes* was the dominant phylum in the AH-nAR group, while *Fusobacteriota* and *Actinobacteriota* were dominant in the AH-AR group (Supplementary Figures 1A,B). At the genus level, among the top 20 differential genera, *Fusobacterium*, *Actinomyces*, and *Veillonella* were significantly increased in the AH-AR group compared to the AH-nAR group (Supplementary Figures S1C,D).

In addition, to identify species with significant differences in abundance between the two groups, we conducted LEfSe analysis using LDA scores > 4.0 and *P* < 0.05. The results showed that the microbial species marked in orange were enriched in AH-AR patients, while those marked in green were enriched in the AH-nAR group (Supplementary Figure S2A). Significant differences in adenoid surface microbiota were observed between the AH-nAR group and AH-AR group across all taxonomic levels from phylum to species (Supplementary Figure S2B). Those results indicated significant differences in the composition of surface microbiota on adenoids between AH-nAR group and AH-AR group.

### Metabolomics analysis

To investigate the effect of AR on AH metabolism, we used a high-resolution LC-MS/MS platform to perform non-targeted metabolomics analysis of metabolites in surface secretions of adenoids. Out of a total of 18 samples, 706 metabolites were identified. Partial least squares discriminant analysis (PLS-DA) was used to classify the samples (Figure 2A). PLS-DA showed that the AH-nAR patients and the AH-AR group samples were significantly separated. 255 metabolites with significant changes were screened under VIP > 1, log2fc > 0.4, and *P* < 0.05 conditions (Supplementary Table S2). Visualize differential metabolic patterns using volcano maps (Figure 2B) and heatmaps (Figure 2C).

**Figure 2:**
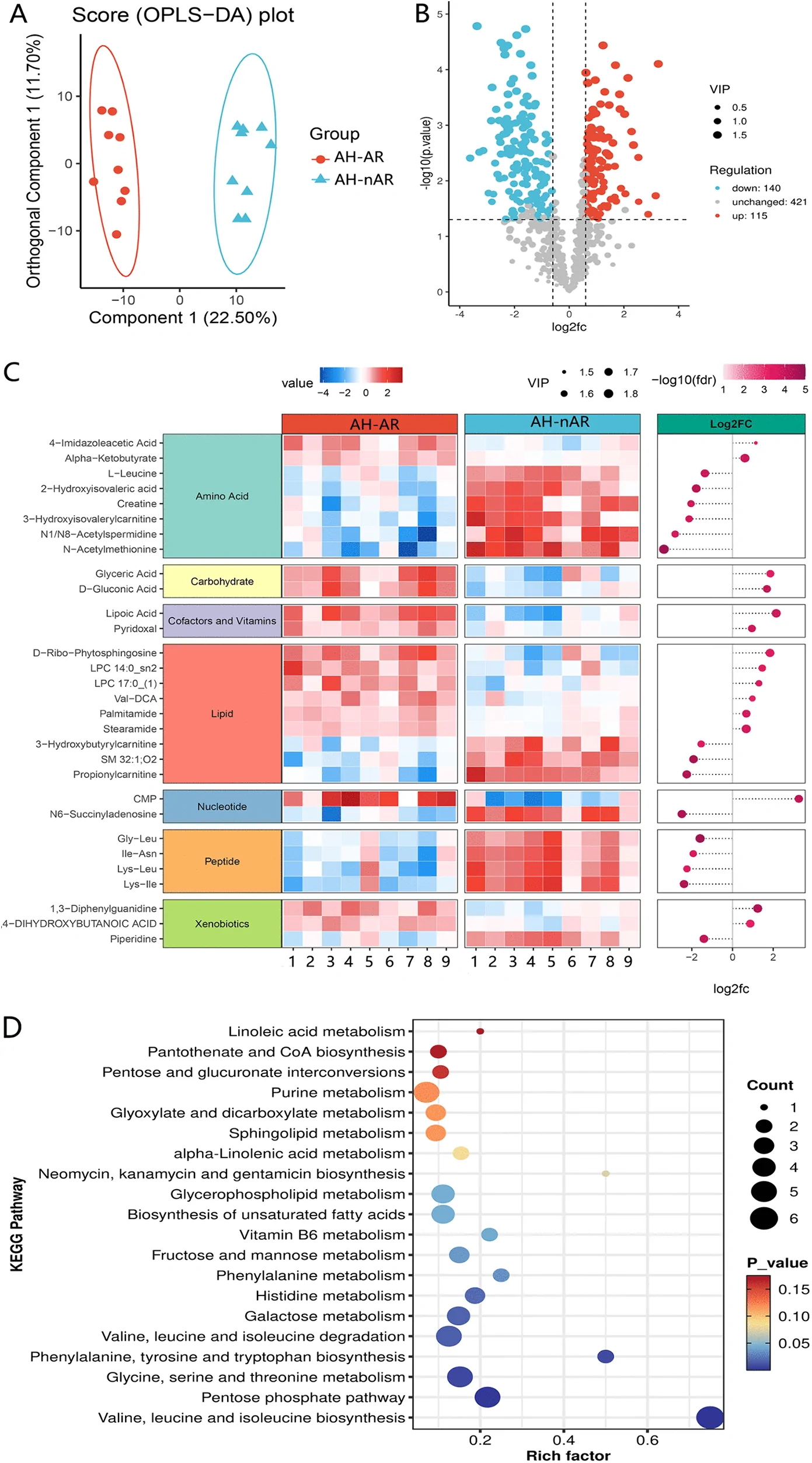
Non–targeted metabolomics analysis of adenoid surface metabolites in the AH–NAR group and the AH-AR group. **(A)** Classification of the samples from the two groups using PLS-DA; **(B)** Visualization of differential metabolomics analysis of the two groups using volcano maps; **(C)** Visualization of differential metabolomics analysis of the two groups using heatmaps; **(D)** Prediction and analysis of the functions of microbiota using KEGG pathways.

To explore potential functional differences between the AH-nAR and AH-AR microbiota, we predicted and analyzed the function of microbiota through the KEGG pathway. The results showed that compared with the AH-nAR group, the AH-AR group exhibited significant increases in the following metabolic pathways, including Valine, leucine and isoleucine biosynthesis, Pentose phosphate pathway, Glycine, serine and threonine metabolism, Phenylalanine, tyrosine and tryptophan biosynthesis, Valine, leucine and isoleucine degradation, Galactose metabolism, Histidine metabolism, Phenylalanine metabolism, Fructose and mannose metabolism, Vitamin B6 metabolism, Biosynthesis of unsaturated fatty acids and glycerophospholipid metabolism (Figure 2D).

### Correlation between the surface microbiome of adenoids and metabolites

Using multi-omics analysis to investigate the correlation between the surface microbiome of adenoids and metabolites, we calculated Spearman correlations between 3 differential genera and 30 differential metabolites (Figure 3). The figure showed that *Fusobacterium*, *Veillonella*, and *Actinmyces* are positively correlated with 11, 10, and 14 metabolites, respectively. In addition, *Fusobacterium*, *Veillonella*, and *Actinmyces* were negatively correlated with 5, 2, and 11 metabolites, respectively.

**Figure 3:**
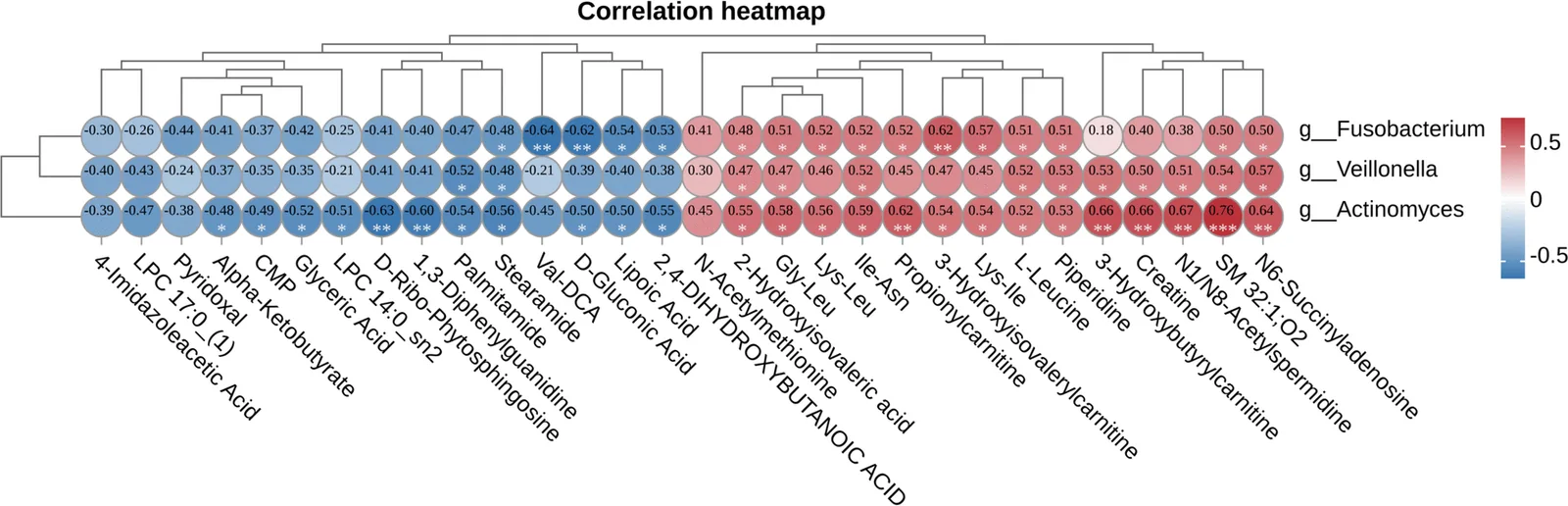
Using multi-omics analysis to explore the correlation between the microbial community and metabolites on the surface of adenoids.

## Discussion

The adenoids are located at the posterior end of the nasal cavity, with severe AH tissue adjacent to or even adjacent to the nasal mucosa. AR and AH are both important components of the upper respiratory tract, and they may interact with each other (19). Research has found that mast cells in adenoid tissue can bind to specific IgE, and their levels of specific IgE antibodies are significantly higher than serum levels (20). The number of eosinophils in serum and adenoid tissue of children with AH complicated with AR is significantly higher than that of children with simple AH. AR symptoms are positively correlated with the level of eosinophils in adenoids (21). AH may lead to the accumulation of allergens such as dust and pollen in the nasal cavity and nasopharynx, thereby exacerbating AR symptoms and their duration (22). Our clinical data shows that the tIgE and rTNSS of AH children complicated with AR are significantly higher than those of simple AH children, and the number of microbial species on the adenoid surface of children with AH complicated with AR is significantly higher than that of children with simple adenoidal hypertrophy. It suggested that the occurrence and development of AH are closely related to AR.

So far, microbiota has become a new tool for preventing, diagnosing, and treating upper respiratory and upper gastrointestinal diseases such as AR, AH, tonsillar hypertrophy, and periodontal disease. Compared to traditional diagnostic and therapeutic methods such as endoscopy and surgical treatment, bacterial biomarkers may be a new approach for diagnosing and treating AH. The non-cultured molecular method based on 16S rDNA sequencing can be applied to studying microbial communities on the surface of adenoids and within tissues. Liu et al. found through 16S rRNA gene sequencing that the abundance of *Bacteroides*, *Faecalibacterium*, and *Ruminococcus gnavus* increased in the feces of AH patients, with *Bacteroides* maintaining a stable increase with age (23). Therefore, it is believed that the gut microbiota disorder is related to AH. Johnston et al. (24) detected *Bacteroides* and *Streptococcus* in glandular crypts, observed *Fusobacteria*, *Pseudomonas*, and *Burkholderia* in adherent bacteria, and *H. influenzae* in tissue epithelium. However, there is currently no consensus on the types of microbiota in the nasopharynx and their relationship with AH. In this study, we identified 1,503 microbial genera in the nasopharyngeal microbiota of children with AH, among which 284 genera were shared between the AH-nAR group and the AH-AR group.

Children's immune function is relatively incomplete, and adenoids are prone to becoming parasitic sites for bacteria and viruses. Bacterial infection is currently considered the main cause of AH and its recurrent episodes in children. At the same time, many microorganisms reside on the surface of the adenoids, which stimulate the immune response of the adenoid tissue and may be the leading cause of AH (25). Brook et al. identified through bacterial culture that the aerobic bacteria in pediatric adenoid tissue were predominantly *Streptococcus*, *Haemophilus*, *Staphylococcus*, and *Moraxella*, while the anaerobic bacteria were mainly *Peptostreptococcus*, *Prevotella*, and *Fusobacterium* (26). As the gateway to the respiratory tract, the nasal cavity and nasopharynx are interconnected. We hypothesize that their resident microbiota may be closely related. Our results indicate that there are significant differences in the taxonomic composition and abundance of various bacteria in the nasopharyngeal microbiota of AH patients with or without AR at the phylum and genus levels, and there are significant differences in the surface microbiota of adenoids. Jensen et al. (27) identified 15 microbial communities in the nasopharynx using 16S rRNA sequencing, including *Fusobacterium*, *Actinmyces*, and *Veillonella*. Our study showed that, compared to children with AH, those with AH complicated with AR exhibited *Fusobacterium*, *Actinomyces*, and *Veillonella* as dominant bacterial genera on the adenoid surface. These results suggest that AR can cause significant changes in the surface microbiota composition of adenoids in AH patients.

The impact of microbiota on the pathology of other organs is mainly mediated by bacterial metabolites, which may affect the immune response in distant parts of the body. We screened a total of 706 metabolites on the surface of adenoids in children with AH with or without AR, among which 255 metabolites showed significant changes. Compared with children with simple AH, those with AH complicated with AR showed significant enrichment in 12 KEGG pathways. Additionally, in children with AH complicated with AR, *Fusobacterium, Veillonella*, and *Actinomyces* exhibited positive or negative correlations with multiple metabolites. These findings suggest that AR may increase the abundance of *Fusobacterium*, *Actinomyces*, and *Veillonella* in the nasopharynx, activate multiple metabolic pathways, and thereby alter the metabolic products of the nasopharyngeal microbiota, potentially exacerbating the risk of AH in children.

There are some limitations to this study: firstly, it is not a community-based study and only includes severe pediatric patients who seek treatment at tertiary medical centers; Secondly, the specific relationship between nasopharyngeal microbiota and metabolites needs to be further confirmed through additional experiments, such as microbiota transplantation; Thirdly, due to limited funding, the clinical sample size of this study is relatively small, and future multi-center large-scale sample studies will be required.

In this study, we characterized the composition and function of the nasal pharyngeal microbiota in children with AH with or without AR. We also identified three nasopharyngeal bacterial biomarkers with strong diagnostic potential in distinguishing AH with or without AR. Although these results require further validation, they may also provide new therapeutic approaches for treating children with AH affected by AR.

## Data Availability

The original contributions presented in the study are publicly available. The data presented in the study are deposited in the (National Center for Biotechnology Information) repository, accession number PRJNA1542836.

## Supplementary material

Supplementary Figure S1 **The differences in the phylum and genus levels of the adenoid surface microbiota between the AH-nAR group and the AH-AR group.** (A, B) The differences in the phyla of the microbiota between the two groups; (C, D) The differences in the genera of the microbiota between the two groups. *, P < 0.05. **, P < 0.01.

Supplementary Figure S2 **The differences in the enrichment of microbial species between the AH-nAR group and the AH-AR group.** (2A) The microbial species marked in orange are enriched in AH-AR patients, and the microbial species marked in green are enriched in the AH-nAR group; (2B) There are significant differences in the adenoid surface microorganisms from the phylum to the species level between the AH-nAR group and the AH-AR group.

Supplementary Table S1
Comparison of Various Clinical Indicators of recruited participants.

Supplementary Table S2
Combined DEMs list.
