# Supplementary material for: The impact of allergic rhinitis on nasopharyngeal microbiota and metabolomics in children with severe adenoid hypertrophy
Source: Front Med (Lausanne). 2026 Oct 15;13:1895589. doi: 10.3389/fmed.2026.1895589 (PMC13717848; doi:10.3389/fmed.2026.1895589)
Supplement: Supplementary file 1 [file Table1.docx]

#### Supplementary Table 1 Comparison of Various Clinical Indicators of recruited participants

| Clinical indices | Recruited participants | | t/χ2 index,  *P* value |
| --- | --- | --- | --- |
|  | AH-AR(n=9) | AH-nAR(n=9) |  |
| Sex (Male, Female) | 5, 4 | 4, 5 | 0.22, 0.64 |
| Age | 7.78±1.92 | 7.11±2.85 | 0.58, 0.57 |
| BMI | 15.47±2.0 | 16.73±1.70 | 1.44, 0.17 |
| CBC+CRP |  |  |  |
| Red blood cell-related parameters |  |  |  |
| RBC | 4.78±0.26 | 4.52±0.49 | 1.43, 0.17 |
| Hb | 126.20±14.31 | 131.30±18.71 | 0.65, 0.52 |
| HCT | 39.64±3.01 | 40.62±2.47 | 0.75, 0.46 |
| MCV | 85.99±4.61 | 87.28±4.93 | 0.57, 0.57 |
| MCH | 28.09±1.68 | 28.37±1.459 | 0.37, 0.71 |
| MCHC | 337.10±11.09 | 330.80±10.16 | 1.26, 0.23 |
| RDW | 13.55±1.05 | 13.45±1.10 | 0.20, 0.84 |
| White blood cells-related parameters |  |  |  |
| WBC | 8.417±2.472 | 7.443±1.674 | 0.98, 0.34 |
| N | 55.27±6.107 | 55.78±8.097 | 0.15, 0.88 |
| ANC | 4.017±0.99 | 3.672±0.6797 | 0.86, 0.40 |
| L | 42.02± 11.05 | 40.70±12.06 | 0.24, 0.81 |
| ALC | 4.77±1.10 | 4.74±0.73 | 0.08, 0.94 |
| M | 5.578±2.59 | 7.07±1.39 | 1.52, 0.15 |
| AMC | 0.49±0.25 | 0.55±0.20 | 0.62, 0.54 |
| E | 4.60±2.67 | 5.93±1.96 | 1.21, 0.24 |
| AEC | 0.31±0.23 | 0.3433±0.1591 | 0.38,0.71 |
| B | 0.42±0.32 | 1.08±1.45 | 1.32, 0.21 |
| ABC | 0.04±0.02 | 0.03±0.04 | 0.59, 0.56 |
| Platelets-related parameters |  |  |  |
| PLT | 375.40±56.36 | 329.00±80.95 | 1.41, 0.18 |
| MPV | 9.81±1.12 | 9.71±0.9714 | 0.20, 0.84 |
| PCT | 0.18±0.06 | 0.20±0.05862 | 0.68, 0.51 |
| PDW | 12.45±2.37 | 12.50±2.068 | 0.05, df=16 |
| CRP | 2.13±1.41 | 2.92±1.523 | 1.13, 0.97 |
| tIgE | 117.00±143.50 | 1225.00±776.10 | 4.21, 0.00 |
| rTNSS | 5.00±1.41 | 9.11±1.45 | 6.08, 0.00 |
